# Artificial intelligence in physical education: comprehensive review and future teacher training strategies

**DOI:** 10.3389/fpubh.2024.1484848

**Published:** 2024-11-08

**Authors:** Yuping Wang, Xinyan Wang

**Affiliations:** ^1^College of Philosophy and Society, Jilin University, Changchun, China; ^2^College of Physical Education, Jilin University, Changchun, China

**Keywords:** artificial intelligence, physical education, teacher training, public health, comprehensive review

## Abstract

Artificial intelligence (AI) technology is deeply changing our lives and provides impetus for improving production and living efficiency as an important emerging tool. Digitalization and intelligent development have also become the development direction of the sports industry, bringing new requirements to the transformation of physical education (PE) and the improvement of the quality of PE teachers. PE is an important part of the public health system, and AI can deeply participate in the formulation of teaching strategies, the tracking of teaching processes and the evaluation of teaching results, effectively improving the quality of teaching. Research on the application of AI technology in PE has been carried out. This paper comprehensively reviews the existing research and conducts a comprehensive analysis of the research progress and status. The potential application areas of AI in PE are discussed to better promote the intelligent and digital upgrading of PE. We found that the research on the application of AI in PE is still in its early stages, and the research content needs to be strengthened in terms of breadth and depth. Furthermore, this paper analyzes the challenges faced by PE teacher development and training in the context of educational transformation in the era of AI, and explores the necessary skills and knowledge related to AI technology that future PE teachers should master in order to effectively achieve the improvement of teaching level and the sustainable development of public health system. The review of this paper provides valuable guidance for educators and policymakers to formulate high-quality teacher development and training mechanisms, and provides a new reference for the application and development of AI in sports.

## 1 Introduction

Physical education (PE) is an important part of the public health system, which plays an important role in improving students' physical and mental health. By teaching and organizing students to participate in various sports, students' sports skills can be cultivated, their physical fitness can be enhanced, and their teamwork spirit and competitive awareness can be improved. Experiments have shown that moderate-intensity and high-intensity exercise have great benefits for students' health (1). At the same time, PE is also committed to cultivating students' psychological qualities such as self-confidence, perseverance and resilience, helping them cope with various challenges in learning and life. In the long run, cultivating students' good exercise habits and lifelong exercise awareness through PE teaching is of great significance to improving the overall health level of society (2).

Although PE plays an important role in the all-round development of students, traditional PE faces many challenges in practice. First, the teaching process usually uses the same teaching plan for all students, ignoring the differences between individual students (3, 4). In fact, different students not only have different physiques, but also have different interests in sports events and methods. It is difficult for traditional large-class teaching to provide more appropriate guidance and help for students' characteristics. Secondly, the traditional sports evaluation mechanism is relatively simple and lacks a scientific evaluation system and data support. The teaching method is relatively single and the classroom organization process depends on the subjective decision of the teacher, which requires high experience and ability of the teacher, and there are obvious differences in the quality and ability of the teacher. The existing physical fitness evaluation mechanism depends on the test results of fixed physical fitness items, which may not fully reflect the overall quality of the students (5). In addition, the shortage of high-level teachers in remote areas has affected the teaching quality (6), and PE is also facing the problem of unbalanced teacher ratio. At the same time, cultivating students' good exercise habits is of great significance to their lifelong health, but traditional teaching methods make it difficult to track the exercise process of all students in detail, and it is even more difficult to conduct further personalized analysis and improvement.

**Figure 1:**
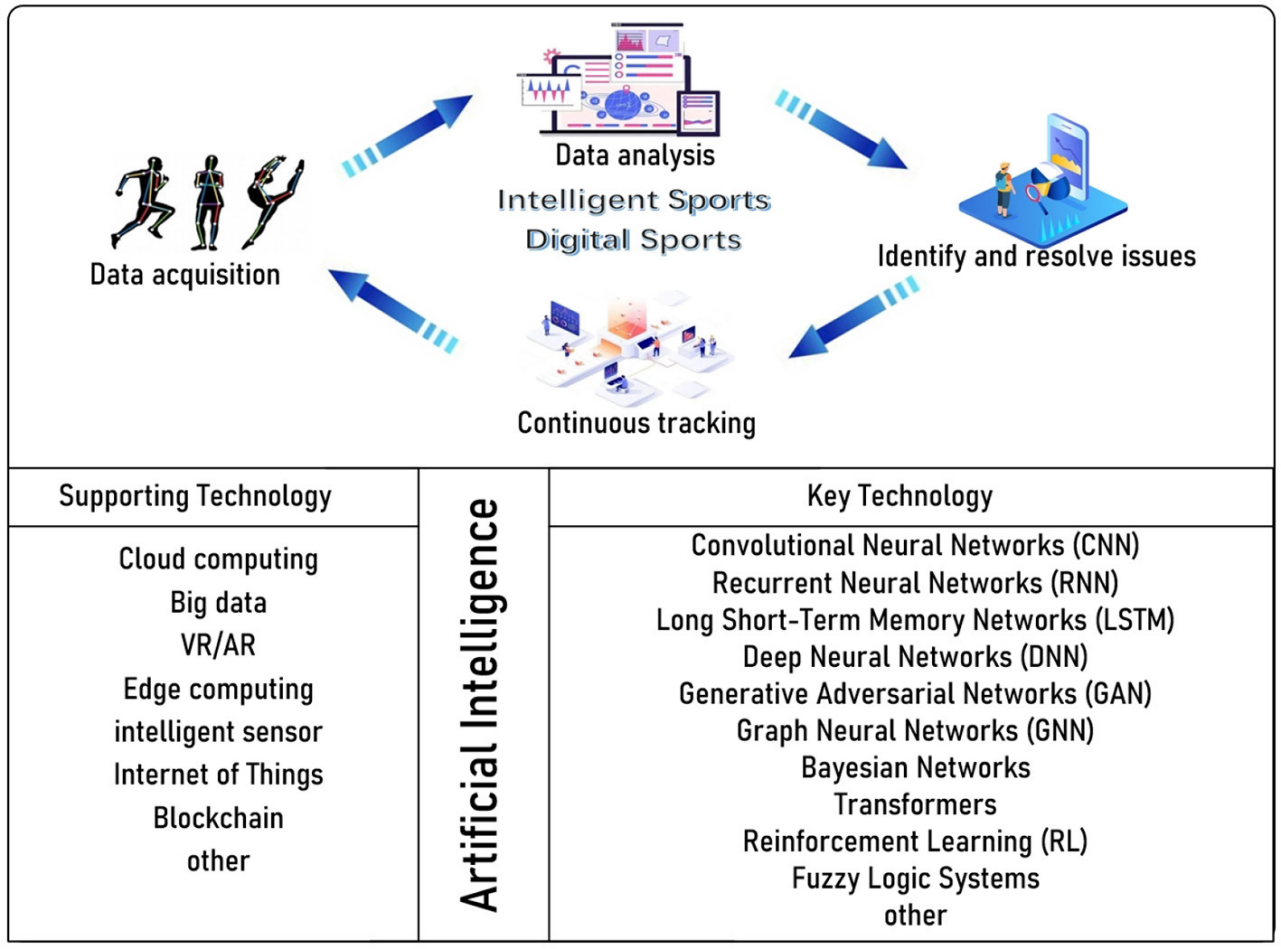
AI in intelligent and digital sports.

The rapid development of artificial intelligence (AI) technology provides a good solution for optimizing the mechanism of PE. In recent years, AI has gradually penetrated into various fields (7), including education. With the penetration of AI technology into the field of education, AI technology has brought new possibilities for the innovation of traditional education through its powerful data processing and intelligent analysis capabilities. As shown in Figure 1, AI technology is the key support for smart sports and digital sports. Its supporting technologies mainly include cloud computing, big data, VR/AR, edge computing, intelligent sensor, internet of things and blockchain, etc., which can help realize the identification, collection, transmission, storage and encryption of key data, and provide basic processable data sets for AI models. AI's key technologies include Convolutional Neural Networks (CNN), Recurrent Neural Networks (RNN), Long Short-Term Memory Networks (LSTM), Deep Neural Networks (DNN), Generative Adversarial Networks (GAN), Graph Neural Networks (GNN), Bayesian Networks Transformers, Reinforcement Learning (RL) and Fuzzy Logic Systems, etc., which can analyze the potential rules in PE based on basic data sets, complete data analysis, classification, clustering and prediction, assist schools, teachers or students to optimize teaching plan design, effect evaluation, injury warning and other applications, and improve the overall teaching effect of PE. In the future, AI technology will run through the development of digital intelligent PE, and data acquisition, data analysis, identification and resolution issues, and continuous tracking will form a good closed loop of AI technology analysis, research, and optimization.

With the gradual deepening of the application of AI technology in PE, more and more research and practice have shown that AI can play an important role in improving the effectiveness of PE teaching, optimizing the teaching process, and promoting student development. Through AI technology, teachers can obtain detailed student sports data, conduct accurate analysis, develop personalized training plans, and improve the pertinence and effectiveness of teaching. At the same time, AI technology can simulate real sports scenes through virtual reality (VR) and augmented reality (AR), helping students train in a safe environment and improve sports skills. In addition, AI technology can also evaluate students' sports performance in real time, provide instant feedback, and help students improve quickly. These applications not only improve the quality and effectiveness of PE, but also stimulate students' interest and enthusiasm in sports.

Although AI technology has shown great potential in PE, PE teachers at this stage still face many defects and challenges when handling this emerging technology (8). First, many PE teachers lack basic knowledge and usage skills of AI technology, and it is difficult for them to effectively apply AI tools in teaching. Secondly, training on AI technology in the traditional teacher training system is insufficient, and it is difficult for teachers to master complex AI technology through self-learning. In addition, some teachers are resistant to AI technology and are worried that AI will replace their teaching work. This mentality affects the promotion and application of AI technology. Therefore, in order to give full play to the role of AI technology in PE, it is urgent to incorporate the learning and application of AI technology into the training process of PE teachers to improve their acceptance and application ability of AI technology.

In order to better promote the development of AI in PE and improve the ability of PE teachers, this paper systematically reviews and discusses the existing research related to AI used PE. In order to fully understand the current research status of AI applications in PE, identify the shortcomings of the research, and explore future research directions. We reviewed the articles that were retrieved by SCIE or SSCI and whose research content was to use AI methods in PE. The keywords for literature search included but were not limited to the following combinations: “artificial intelligence,” “deep learning,” “machine learning,” “physical education,” “application of AI in education,” “digital sports,” “intelligent educational tools,” etc. In order to maintain the focus of the literature review, AI application research not related to physical education and articles that had been retrieved but retracted were excluded. By analyzing the current status of AI applications in PE, the challenges it faces, and future development directions, we aim to provide valuable references for educators and policymakers, promote the widespread application of AI technology in PE, and provide guidance for cultivating high-level PE teachers who can adapt to new situations and technologies. We hope that this review will promote the deep integration of AI technology and PE, improve the overall level of PE, contribute to the all-round development of students, and help improve the health level of the whole people.

## 2 Literature review

### 2.1 Applications of AI in PE

In the field of PE, the application of AI technology is developing rapidly, making PE more interactive and targeted, and improving students' participation and learning effects. AI technology is mainly used for assistance in the PE teaching process, evaluation of students' learning effects, design of personalized teaching, and classroom interaction. Its core goal is to improve students' learning interest and exercise effect, which can also help teachers optimize teaching strategies.

Emerging AI technology products can become auxiliary tools for PE, helping to improve the level of PE. Keiper et al. (9) explored the feasibility of using ChatGPT to assist sports management education, and believed that ChatGPT can become a versatile tool to help teachers and students complete various tasks in PE. The authors also discussed the issues that need to be paid attention to when using ChatGPT, and put forward practical suggestions on how ChatGPT can supplement and support sports management education.

The development of information technology has provided new feasibility for the expansion of sports teaching mode. Shin et al. (10) built a virtual sports classroom based on AI technology, where teachers can supervise and guide students' sports process online. AI based posture detection technology is applied to the system, and five interesting games are designed to increase students' interest in participation and the effectiveness of exercise. Although the system is designed to enable students' PE classes during the COVID-19 pandemic, various factors such as severe weather and students' negative emotions may also prevent students from effectively participating in offline PE classes (11), and online virtual classes are a useful supplement to offline classes. Wang et al. (12) also proposed a virtual reality PE teaching and learning environment for college and university students. Support vector machine (SVM) and particle swarm optimization (PSO) technologies are used to accurately classify and evaluate students' performance in a range of physical activities on the basis of interactive and immersive VR content generated by the hardware layer.

Too many evaluation factors and insufficient evaluation framework have brought certain challenges to the evaluation of PE teaching in colleges and universities. In order to simplify the evaluation process and provide an effective framework for evaluating teaching methods, Li et al. (13) studied a multi-feature fuzzy evaluation model based on artificial intelligence. The framework uses fuzzy instructions to integrate natural/human language, considers three evaluation perspectives, including management stage, teachers and students, and adopts an enhanced cuckoo search optimization algorithm. It combines the student flow mechanism and motion vector deconstruction designed based on functional standards.

When teaching PE to people with different physical constitutions, it is difficult to achieve global physical health optimization with a unified exercise method. In order to provide more high-quality, customized and personalized health management services based on the physical characteristics of the population in the process of PE and guidance, Li and Li (14) used the Bamberg algorithm to optimize the Back Propagation Neural Network to provide decision-making for the selection of exercise prescriptions for people with different physical constitutions. The system is trained based on a large amount of historical data and experience summary to improve the flexibility and accuracy of providing reliable prescriptions for users with different physical constitutions.

In the teaching process, in addition to setting up flexible teaching plans for students with different physical conditions, it is also very important to monitor and evaluate the physical condition of each student during the learning process, which requires reliable technical support. Feng (15) proposed a sports management system based on AI, which mainly completes the management of students' sports performance and the evaluation of their physical condition. Based on a large amount of data such as physical fitness and sports performance generated by students in the process of PE courses, the C4.5 algorithm is used as a decision tree classification algorithm for data mining, the data is divided into multiple sub-data sets, and a performance management and physical fitness analysis system based on data mining is further constructed.

It is necessary to establish a scientific and systematic school sports environment evaluation system and a good classroom teaching effect evaluation mechanism. IoT and AI technologies are important supports for the comprehensive evaluation system. Yu and Mi (16) built a sports teaching system based on IoT. The golden sine algorithm (Gold-SA) and back-propagationneural network (BPNN) model are used to evaluate the proposed model. Based on this system, teachers can publish learning content with 3D demonstration capabilities to students and can also remotely observe students' sports conditions.

In addition to improving students' athletic performance, cultivating good exercise habits to ensure their long-term physical health is also a very important teaching goal. Traditional classroom teaching methods still have shortcomings in monitoring and evaluating students' exercise habits. Peng and Tang (17) proposed a comprehensive intelligent service system, including an intelligent terminal device platform, a stadium intelligent platform, and a health cloud management platform. Text and audio format data of the teaching process are collected and stored, and AI is used for background data analysis and processing to promote long-term supervision and comprehensive analysis of the classroom teaching process.

In addition to researching new methods and technologies for improving students' abilities, it is also very important to improve the teaching level of PE teachers. Teaching methods and concepts need to adapt to the development of technology and the needs of students. Cao et al. (18) designed an intelligent PE tracking system to comprehensively evaluate students' learning process and teachers' teaching effectiveness. The system integrates technologies such as virtual reality, intelligent recognition, big data analysis, fuzzy-level evaluation and machine learning. It collects students' sports data in PE classes through IoT, and can replay the sports process through virtual reality based on the data on the human-computer interaction terminal. It can also comprehensively evaluate teachers' teaching courses based on evaluation algorithms to promote the comprehensive improvement of PE teaching ability.

Mental health problems are hidden and easily overlooked. Psychological health education for sports students is also a teaching content that needs attention. Liang et al. (19) used big data and AI to evaluate the mental health education of college students majoring in sports. The educated, mental health information, and the receiving intermediary are in an orderly cycle and transform with each other, which together constitute the operation mechanism of college students' mental health big data model, and CNN is used for early warning of students' mental health problems.

In the process of promoting the development of digital smart sports led by technologies such as IoT and AI, the ability, willingness and quality of teachers are also very important. Bucea-Manea-Toniş et al. (20) studied the experience and status of teachers when using information intelligent teaching methods. The survey results show that although the overall results of online teaching are good, students and teachers will have certain psychological pressure. Teachers are satisfied with the online teaching methods and platforms, their teaching ability and work level are improved, and they believe that MOOC platforms and VR applications are good online teaching solutions in experiential and game-based teaching.

The improvement of PE infrastructure is also of great significance to optimizing teaching effect. With the continuous improvement of informatization, more data information needs to be transmitted through the network. Zhai and Chen (21) used machine learning algorithm to preprocess and extract features of relevant data in the optical communication system of traditional PE information system, and further selected appropriate machine learning model for training and optimization, and finally applied it to the optical communication system to improve communication rate and ensure teaching effect.

### 2.2 Applications of AI in sports training teaching

The application of AI in sports training and management provides athletes and coaches with more accurate and effective tools. Through IoT devices and deep learning technology, AI can monitor athletes' physical condition and performance in real time, thereby optimizing training plans and management strategies. This technology not only improves the competitive level of athletes, but also improves the safety and scientificity of the training process.

In the traditional sports training and learning process, students need to rely on the experience of teachers and coaches to solve problems they encounter. Face-to-face teaching is not only not flexible enough, but also difficult to ensure the quality of guidance students receive when teachers' abilities are uneven. Wei et al. (22) built a sports theory expert system based on AI technology to help students obtain systematic theoretical and technical support, better learn sports training methods, and promote the improvement of training efficiency. The proposed expert system includes student module, coach module, information management module, knowledge base module, information processing module and teaching module. The proposed system can also analyze the status of students based on the collected information, providing reference for coaches to develop reasonable training plans.

The rapid development of sensor and Internet of Things technology has provided a technical foundation for the digitization and scientificization of sports training. Reliable data of human movement can be sensed, collected and transmitted to the cloud for comprehensive processing by the server. Zhang et al. (23) introduced a system architecture that uses a wearable wireless sensor network to collect student movement data and transmits it to the server based on IoT. Bayesian deep classifiers and deep learning algorithms using adaptive optimization are used to analyze the data collected on the server. The AI algorithms processes the individual's sports behavior and health status data in real time, and complete the prediction of the individual's future status, providing a reference for the student's sports training process and promoting the formulation of personalized and more effective training plans.

The degree of completion during sports training is directly related to the training effect. When teachers have limited energy, using AI technology to identify students' sports training completion is a potential option. Quyang (24) studied the evaluation method of sports training completion based on deep residual network. The collected student action images are preprocessed based on spatial scale filtering and regression factors, and a deep residual network is constructed. Through offline training, the implicit relationship between the athlete's state and the dynamic change process of sports training behavior is understood. In the online application process, the preprocessed action images will be input into the trained evaluation model to evaluate the completion of the athlete's sports training actions in real time. The experimental results show that the evaluation of sports completion based on the processed images has better results.

On the basis of building real-time perception of students' sports status, it is very necessary to design an efficient sports risk assessment and injury warning processing mechanism as injuries will seriously affect students' competitive strength and physical health. Guangde (25) proposed a PE and emergency response system using deep learning to provide first aid protection for students injured in sports, which can help to develop a better PE environment. Wearable sensors based on wireless self-organizing networks are still important tools for collecting data, including IMU, GPS, magnetometer, gyroscope, and accelerometer sensors. Deep neural networks are used to predict students' sports status and potential injuries.

In some sports teaching scenarios, wearable wireless sensor devices will reduce the efficiency of students' sports training. The sports accident emergency response mechanism based on video data sources can serve as an effective supplement. Leilei et al. (26) proposed a method for real-time detection and analysis of sports accidents based on deep convolutional neural network processing of video stream images collected by cameras. When students are injured, the system mainly analyzes the location, type, severity and cause of the injury. Teachers and schools can understand and obtain detailed information about students' injuries more quickly and further develop treatment plans.

### 2.3 Applications of AI in specific sports teaching

In specific sports, the application of AI technology has begun to show great potential. Through data analysis and machine learning, AI technology can help athletes and coaches optimize technical movements, improve training results, and develop more scientific game strategies. The application of these technologies is not only widely used in professional sports, but also gradually penetrated into campus and amateur sports, improving the overall level of sports.

In order to build a robust, efficient and universal tennis teaching intelligent auxiliary system and provide new perspectives and methods for improving the intelligence of sports teaching mode, Song (27) introduced AI technology into tennis teaching. On the basis of demonstrating the applicability of AI in the tennis teaching process, a comprehensive teaching auxiliary system including expert system, image acquisition system and intelligent language system was proposed. In order to solve the difficulties faced by the auxiliary teaching system in the training and learning process, a framework for learning large-scale fuzzy cognitive maps from time series data was designed based on compressed sensing technology.

Establishing a virtual reality-assisted teaching system that is closer to reality can serve as a more effective tool when guiding students. Li et al. (28) studied strategies to improve the quality of football teaching in the mobile Internet environment through 360-degree panoramic VR football teaching videos authorized by the metaverse based on machine learning and the K-means algorithm under AI. Under the cloud-edge collaborative architecture, the optimized transmission strategy of video streams is studied in detail to improve the hit rate of terminals when requesting resources and reduce the overall load of the system.

When AI technology is introduced into real-time guidance for specific sports, it is necessary to perceive and collect students' movement posture and physiological state information in real time. Liu and Hang (29) proposed an intelligent system suitable for volleyball practical teaching. The system can obtain the relative motion information between the sensor and the object through the change of the optical signal, thereby realizing the detection and monitoring of the object's motion state. The stretch characteristics of artificial muscles and the high sensitivity of optical sensors are used to capture and analyze students' motion data in volleyball training in real time, and the movements are identified and evaluated through machine learning algorithms to provide personalized guidance and feedback for coaches.

**Figure 2:**
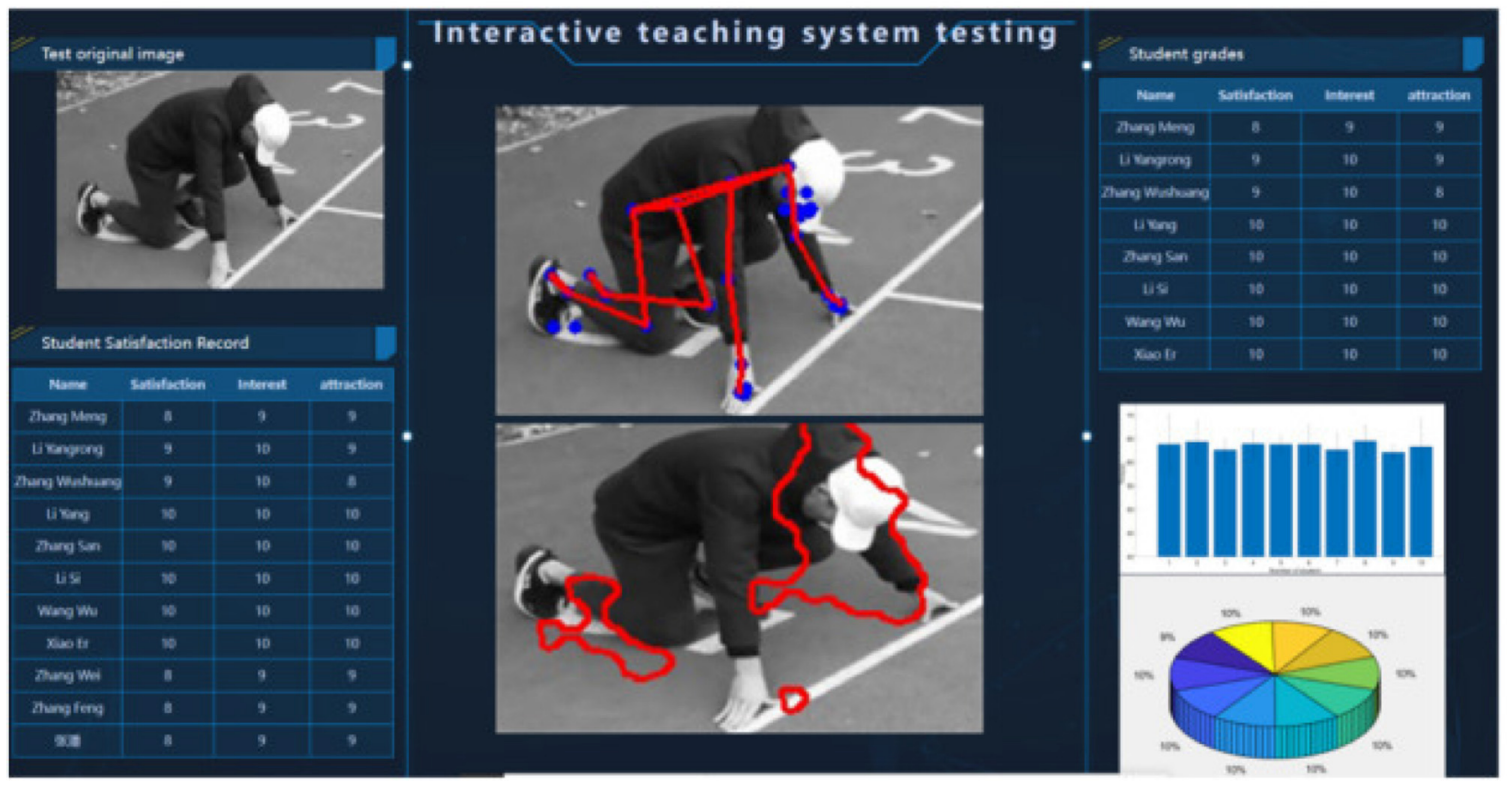
Test result analysis and display interface (30). Reprinted with permission from “Practical Application of Interactive AI Technology Based on Visual Analysis in Professional System of Physical Education in Universities” by Quantao He et al., licensed under CC BY-NC-ND, https://www.sciencedirect.com/science/article/pii/S2405844024006583.

In the traditional teaching process, teachers judge students' sports characteristics and competitive abilities based on their own senses, relying on teachers' accumulated experience rather than a digital scientific system. With the development of AI technology, more scientific, standardized and iterative evaluation tools can be developed. He et al. (30) designed a motion feature extraction system based on Kinectv2 technology and ORB feature extraction algorithm for 400-meter running teaching. The system captures and extracts students' motion posture data in real time, providing real-time dynamic interaction between teachers and students, and promoting the real-time and targeted nature of course teaching. Figure 2 shows the test result analysis and display interface of the system.

The scientific nature of sports training will also have a certain impact on the changes in students' psychological state. Achieving a balanced improvement in physical and mental health is a topic worth studying. Zhang (31) used machine learning technology to clearly identify the impact of different exercise intensities on students' psychological state in basketball, in order to set a reasonable basketball exercise intensity according to the students' conditions, and improve their mental health as much as possible while completing physical exercise.

**Figure 3:**
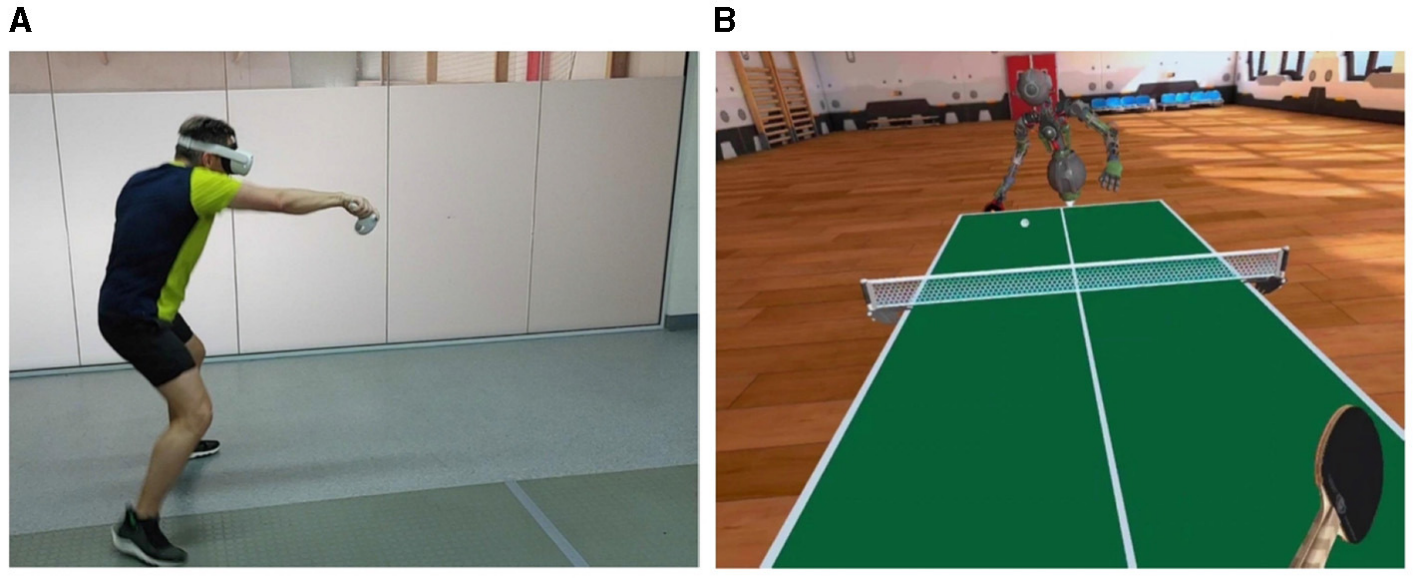
Playing table tennis with AI opponents in VR (32). **(A)** Third-person perspective. **(B)** VR perspective. Reprinted with permission from “Assessment of the Intensity and Attractiveness of Physical Exercise While Playing Table Tennis in an Immersive Virtual Environment Depending on the Game Mode” by Jacek Polechoński, licensed under CC BY 4.0, https://bmcsportsscimedrehabil.biomedcentral.com/articles/10.1186/s13102-024-00945-y.

Students' curiosity about new technologies can motivate them to better integrate into PE based on AI technology. Polechoński (32) used the table tennis VR platform Racket Fury to study the application value of new technologies. Figure 3 shows images of students using the Racket Fury platform to confront AI virtual opponents. The intensity of exercise during training in VR was evaluated by heart rate monitoring and perceived exertion score (RPE 6-20). The effectiveness of training was evaluated based on the user's performance when playing against AI opponents, the user's satisfaction was measured using the Physical Activity Enjoyment Scale, and the potential usefulness of the test application in sports and physical education was evaluated based on a questionnaire of participating physical education teachers (30 participants). The test conclusion shows that competing with AI opponents in VR can increase exercise intensity and sports fun, and physical education teachers highly praised this application.

Despite its broad prospects, the application of AI in PE still needs to go through a process of continuous development and improvement. Masagca (33) used AI to generate a set of training plans for aerobics and a human coach to develop another set of plans. By using different training plans in different student groups, the effects of the two training plans were compared. The results showed that although the plans generated by AI also have certain effects, they are generally not as effective as the training plans developed by human coaches in improving students' quality and ability.

**Figure 4:**
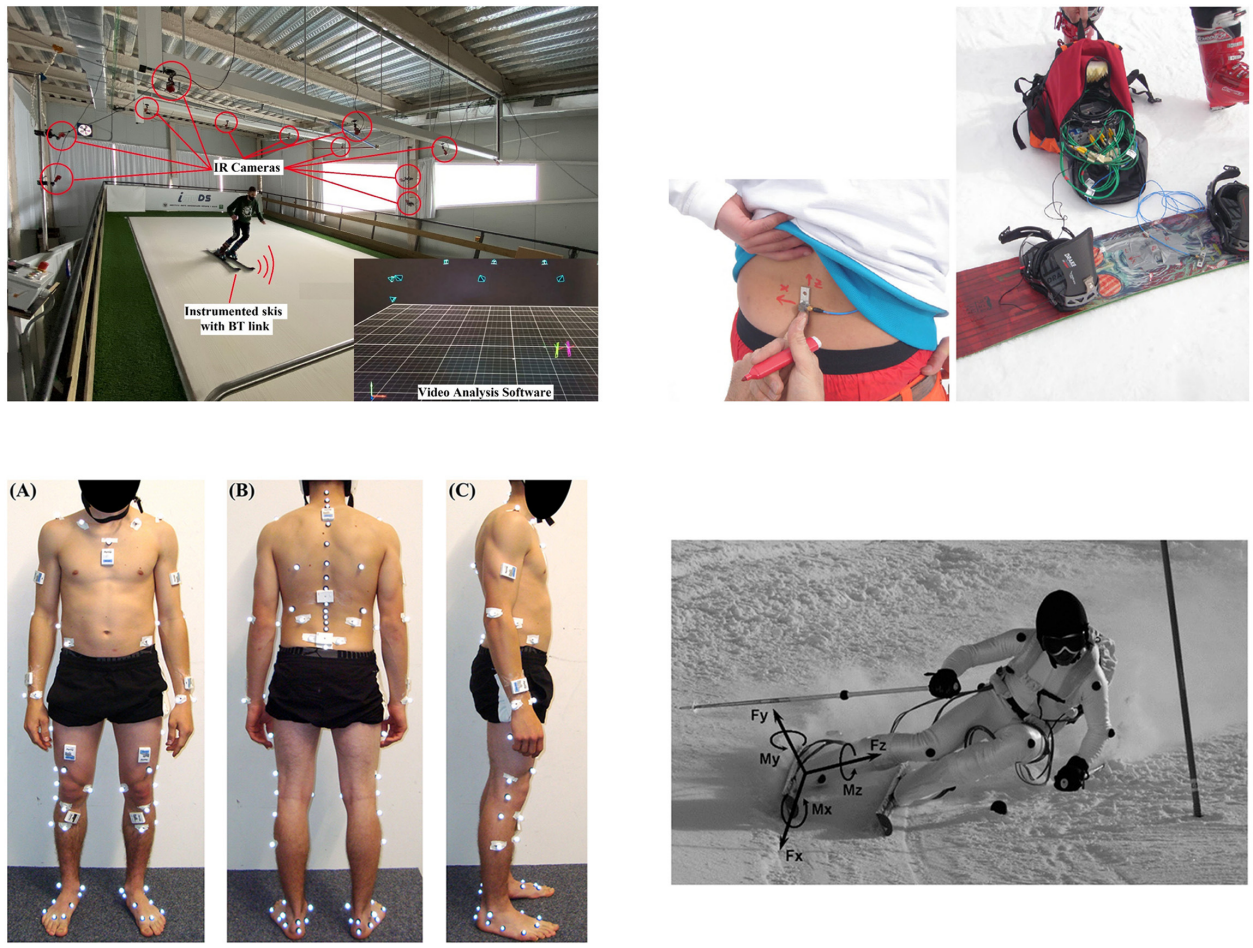
Motion capture technology in skiing. **(A)** Adapted with permission from “Development and Evaluation of a Low-Drift Inertial Sensor-Based System for Analysis of Alpine Skiing Performance” by Isidoro Ruiz-García et al., licensed under CC BY 4.0, https://www.mdpi.com/1424-8220/21/7/2480 **(B)** Reprinted with permission from “Designing, Building, Measuring, and Testing a Constant Equivalent Fall Height Terrain Park Jump” by Nicola Petrone et al., licensed under CC BY 4.0, https://link.springer.com/article/10.1007/s12283-017-0253-y **(C)** Adapted with permission from “An Inertial Sensor-Based Method for Estimating the Athlete's Relative Joint Center Positions and Center of Mass Kinematics in Alpine Ski Racing” by Benedikt Fasel et al., licensed under CC BY 4.0, https://www.frontiersin.org/journals/physiology/articles/10.3389/fphys.2017.00850/full **(D)** Adapted with permission from “Development of a New Embedded Dynamometer for the Measurement of Forces and Torques at the Ski-Binding Interface” by Frédéric Meyer et al., licensed under CC BY 4.0, https://www.mdpi.com/1424-8220/19/19/4324.

It is worth mentioning that there are many new technologies and equipment with great application potential in PE. For example, in skiing, researchers have established a variety of systems to better monitor the athletes' motion posture. Figure 4 shows motion sensing and capture technologies based on infrared cameras (34), IMU sensors (35), visual capture (36), and 3D dynamometers (37). Although these new technologies and equipment for professional sports have not yet been used in PE, and these technologies have not yet been well-integrated with AI to further improve system performance, these technologies can provide a technical basis for the application of AI in PE.

### 2.4 The relationship between AI and PE teachers

In PE, the relationship between teachers and AI is an important research direction. The introduction of AI technology has not only changed the traditional teaching model, but also put forward new requirements for the roles and responsibilities of teachers. Teachers need to work with AI technology and use the tools and data provided by technology to better guide students' learning and training. At the same time, AI technology also provides teachers with more teaching resources and support, improving the overall teaching level.

While studying the optimization of specific PE projects or processes based on AI technology, it is also necessary to explore the relationship between AI and PE from a macro perspective, which is conducive to promoting the comprehensive development of AI technology in the field of PE. Lee and Lee (38) studied the principles and uses of AI technology in the field of PE, and conducted an in-depth analysis of potential applicable technical fields. AI can not only serve as an important auxiliary tool for PE teachers, but also become an effective support for promoting students' independent growth. Through continuous iterative evolution, AI teachers will gradually mature and become more professional. Human teachers need to adapt to the development of technology and learn to use AI tools to improve their teaching ability. Stubbornly doing work that can be achieved by AI will eventually be replaced by AI. Engaging in more creative work is the direction of future PE teachers.

New generation information technologies including IoT, cloud computing, big data, AI and mobile Internet will jointly reshape the model and concept of PE. The process of building a school smart PE (SSPE) system faces many challenges, which requires the joint attention and participation of the government, schools, teachers and students. Deng et al. (39) conducted an in-depth and systematic analysis of the basic theory, construction status, application cases, ecosystem, future challenges and optimization paths of SSPE. Among them, SSPE puts forward higher requirements on teachers' teaching literacy and innovation ability. Teachers need to have more solid information technology application capabilities and be able to use various teaching tools and resources proficiently; at the same time, they need to have keen observation and communication skills, be able to grasp the individual differences of students and realize personalized teaching. Furthermore, they need to have the ability to use data analysis technology in order to find problems in time, adjust teaching strategies and improve teaching effectiveness. Teachers also need to understand the evaluation criteria for SSPE and adjust teaching strategies according to these criteria to improve students' physical performance.

## 3 AI technologies in PE

According to the literature review, we can see that AI technologies have begun to be widely applied in PE. However, the relevant theoretical innovations and engineering applications are not yet widespread and in-depth enough. Fully utilizing AI technology can promote the digital and intelligent development of PE, but this process requires high-level talent teams to plan, research, apply, and validate relevant theoretical frameworks and innovative technologies. In this section, we mainly discuss the AI tools and platforms that can be used in PE, the benefits of AI in PE teaching and learning, and the existing problems in the current development of AI in PE.

### 3.1 AI tools and platforms used in PE

Various AI tools and platforms can be used in PE to improve teaching effectiveness and students' physical and mental quality. These tools and platforms can either be general-purpose commercial solutions or custom-built tools designed specifically for PE needs. Different types of tools have their own advantages and disadvantages, but both of them can enhance the effectiveness of PE instruction and improve management efficiency if use reasonably.

**Figure 5:**
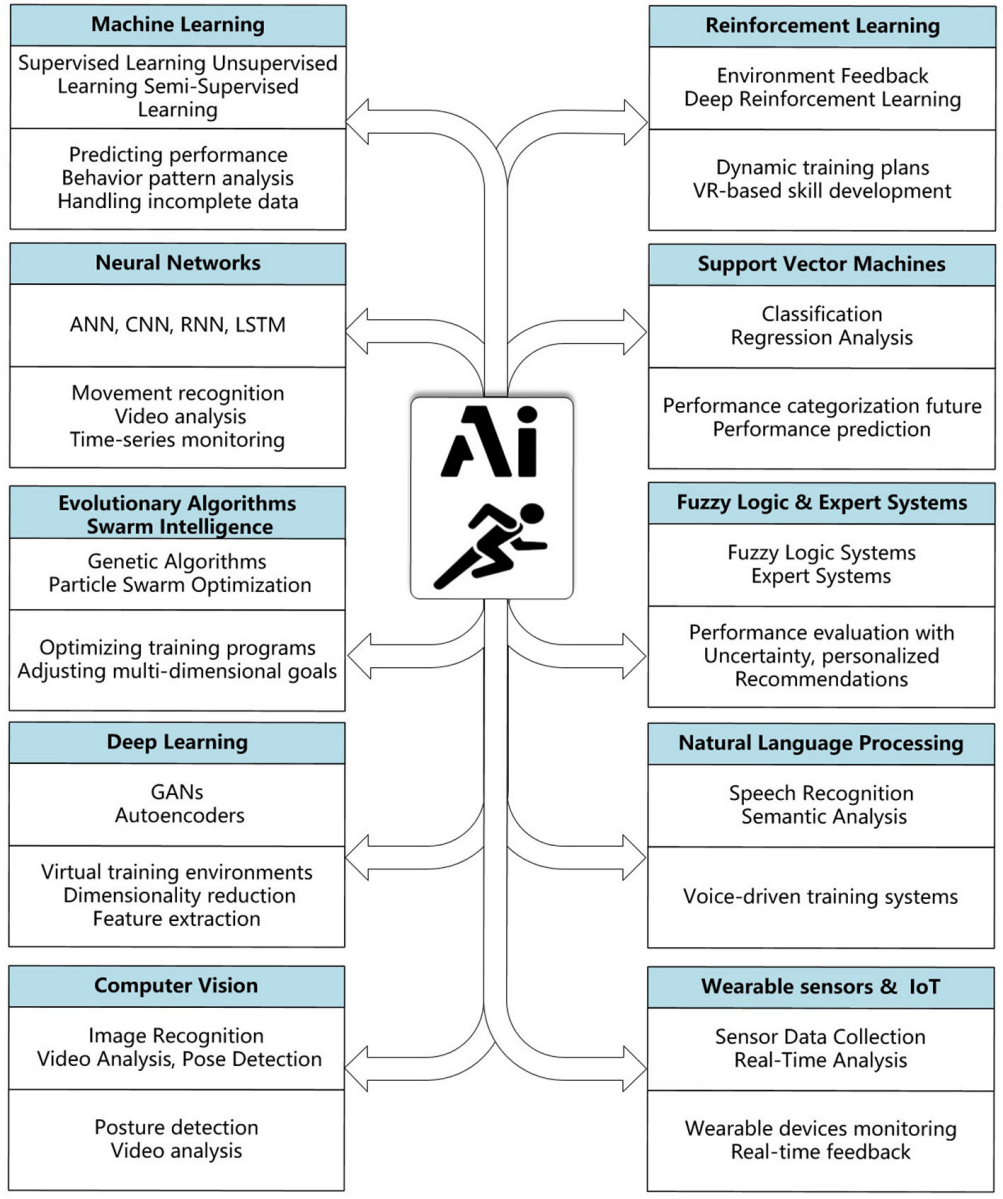
Illustration of potential AI technologies, key technical points, and applications.

From the perspective of technology classification, many AI technologies can be applied to PE, including Machine Learning, Reinforcement Learning, Neural Networks, Support Vector Machines, Evolutionary Algorithms and Swarm Intelligence, Fuzzy Logic and Expert Systems, Deep Learning, Natural Language Processing, Computer Vision, wearable sensors and IOT, etc. Figure 5 shows the key technical points and potential applications of these technologies. (1) Supervised learning of machine learning needs to be based on labeled data, so it is more suitable for some analysis and judgment with deterministic results. For example, if the training data contains two types of data with correct movement postures and incorrect movement postures, the trained model can be used to determine in real time whether the student's movement posture is standard. Since labeling all data requires a lot of manual work, semi-supervised learning improves the model, and only part of the training data needs to be labeled. Unsupervised learning does not require labeling of training data, and is generally used to analyze the potential patterns and laws hidden in the data. For example, analyzing the sports performance trends of student groups or identifying potential associations between different categories of data. (2) Reinforcement learning is a method in machine learning. The process of model optimization requires interaction with the environment, learning how to take actions based on the reward mechanism, and optimizing strategies through continuous attempts. It can be applied to some projects that require interactive learning and continuous improvement. For example, in intelligent training systems, the training plan is continuously adjusted through students' performance and feedback to promote the continuous improvement of system capabilities. (3) Neural networks are also a method in deep learning. They have strong generalization capabilities and can handle nonlinear and complex input data. Therefore, they are widely used in pattern recognition, prediction and classification tasks. Among them, ANN can be used for simple classification and prediction tasks, while CNN is more used to process video and image information and extract local features in images. Time series processing models such as RNN and LSTM can analyze the characteristics of time series data and can be used for tasks such as tracking, prediction and motion pattern recognition in the training process. (4) Support vector machines are mainly used for classification tasks, for example, to classify students' physical condition, sports performance, test results, etc., so as to further customize targeted solutions for their categories. (5) Bionic algorithms represented by evolutionary algorithms and swarm intelligence can be used to optimize and solve complex problems in PE, for example, to find the best balance point under the premise of multiple influencing factors, optimize students' training plans or team formation modes, etc. (6) Models such as fuzzy logic and expert systems are generally used for causal reasoning. Fuzzy logic is generally used to deal with uncertainty and fuzzy reasoning, while expert systems simulate the decision-making process of human experts. When students are learning and exercising in physical education, many related states are also fuzzy, which can be processed using fuzzy logic. Expert systems can simulate teachers or coaches to provide real-time guidance and diagnosis to students. (7) Deep learning is a special form of neural network, which is suitable for automatically extracting features from large amounts of data, and performing classification and regression. It can help teachers classify students so as to provide adaptive guidance and adjust training plans, etc. (8) Natural language processing aims to enable computers to understand, analyze and generate natural language. It can be applied to voice intelligent interaction between teachers and students, provide intelligent interaction modes for other intelligent systems, and automatically generate text information, etc. (9) Computer vision aims to enable computers to understand students' movement status or classroom organization status by analyzing video images, and is an important part of PE intelligence. (10) Wearable devices and IOT are key infrastructures for data collection and transmission in PE. On the one hand, they provide a digital foundation for AI technology, and on the other hand, AI technology can also be used in these infrastructures to improve device performance and user experience.

**Figure 6:**
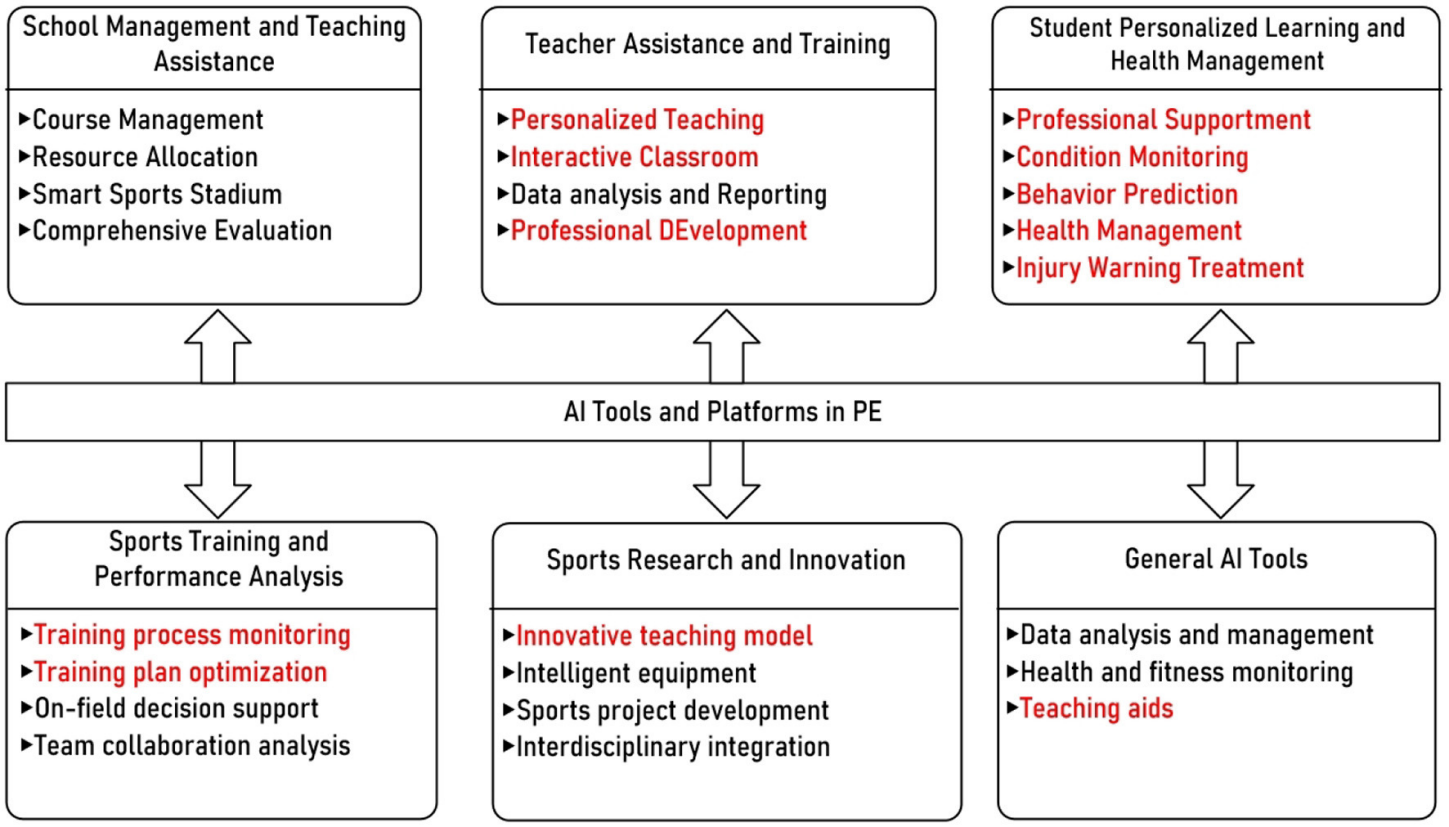
Potential application areas of AI technologies in PE.

Schools, teachers, students and research teams play different roles in PE and have their own needs for AI tools and platforms. As shown in Figure 6, based on research and applications of AI in groups, potential tools and technologies can be mainly divided into six categories from the perspective of application field classification: school management and teaching assistance, teacher assistance and training, student personalized learning and health management, sports training and performance analysis, sports research and innovation, and general AI tools.

AI technology can assist schools in optimizing management and operation models, improve the effectiveness of PE teaching, and promote the improvement of teachers' and students' skills and abilities. In terms of course management, AI technology can help schools better analyze the relationship between different PE course scheduling modes and students' sports skills and physical fitness, ensure the reasonable matching of PE courses with other courses, and improve students' participation and exercise effects. At the same time, by exploring the relationship between different sports projects and the improvement of the quality of students of various physical fitness, the arrangement ratio of various projects in PE courses can be optimized. In terms of resource allocation, facing the dilemma of many classes and students and limited sports venues, AI technology can assist schools in better comprehensive scheduling and promote the efficient use of limited sports resources. According to the characteristics of students in different classes and the existing PE teaching staff ratio, teachers' course arrangements can also be optimized to improve the matching degree between students' needs and teachers' abilities. By establishing a big data platform including various text, audio and video PE materials, AI technology can help schools better push sports exercise consultation and guidance to students. AI technology is also a key support for the construction of smart sports stadium, which can promote the operation efficiency of venues and the experience quality of teachers and students. AI can optimize lighting and HVAC systems based on real-time data, reducing energy consumption while maintaining comfort. Digital sports equipment and intelligent human-computer interaction functions can increase the interest of teachers and students in participating in sports exercises. Comprehensive tracking and analysis of the exercise process can also improve students' sense of achievement and help them optimize their exercise strategies. In terms of Comprehensive Evaluation, it is difficult to evaluate the comprehensive teaching effect of PE in the whole school under the traditional teaching model. AI technology can help schools analyze the teaching characteristics of each teacher and provide recommended guidance for improving teaching ability. The evaluation of students' learning outcomes can provide a basis for the subsequent course setting and adjustment of students. Things are developing dynamically. AI technology can help schools better analyze the dynamic characteristics of teachers and students, and provide impetus for the improvement of PE teaching effect.

For PE teachers, AI will be an important technology for them to implement the teaching process and promote capacity improvement. Considering students with different physical conditions and psychological states, AI technology can be used as data analysis tools to identify each student's strengths and weaknesses, helping teachers design personalized teaching plans. During the execution of these plans, AI can assist teachers in evaluating teaching effectiveness and rescheduling the plans. This promotes the implementation of the concept of tailored teaching and corrects the shortcomings of traditional general teaching models. Meanwhile, AI technology can help teachers better understand the real-time status of students, allowing for flexible adjustments to the classroom pace to better meet students' learning needs, better interactive classrooms can be achieved. Data analysis and reporting require higher capabilities from teachers. They should possess certain research abilities to collect various types of unstructured data according to research objectives and use AI technology to complete data analysis. AI can help teachers explore various potential roles in PE and further help address issues that constrain students' ability improvement. For the teachers' own professional development, AI can help them analyze the teaching effectiveness, providing references for improving their teaching abilities. AI can also recommend personalized training courses and learning resources based on the teachers' backgrounds, teaching experiences, and professional needs, helping them enhance their teaching skills.

As the main participants in PE, students often face numerous doubts and difficulties during class learning and physical exercise due to their lack of knowledge reserves. But AI can provide professional support to them. Models trained with a vast amount of specialized PE knowledge can act as virtual teachers, which can offer flexible, real-time, and efficient knowledge delivery and problem-solving. During physical exercise, students are unable to accurately understand their real-time physical condition and it's also difficult to grasp changes in their long-term health status. AI technology can help students monitor their status, enabling them to better understand their situation, enhance self-regulation abilities, and assist in the design and optimization of long-term exercise plans. Meanwhile, AI technology can predict students' behavior. By using the predicted states based on established teaching or training plans, and comparing them with the actual states collected during teaching, The causes of deviations can be analyzed by AI, providing a basis for further optimization of student learning and training programs. In terms of health management, AI technology can analyze students' health trends based on long-term health status and exercise habit data, helping them adjust exercise planning and develop good health habits. Injury prediction and management are also crucial applications of AI technology. Potential injury risks can be identified by monitoring real-time exercise data such as heart rate, step frequency, and muscle load. For example, if the system detects an abnormally high heart rate or improper movements, AI can warn the students who can adjust their exercise intensity or posture to avoid injuries. Meanwhile, AI can analyze students' historical exercise data to uncover long-standing issues in exercise habits, thus preventing potential injuries. After an injury occurs, AI can assist in analyzing the causes of injuries and recommending treatment and rehabilitation plans to help students recover as soon as possible.

Apart from enhancing physical fitness, an important goal of PE is to improve students competitive skills, enabling them to achieve better performance in competitions. Adaptive research and development of AI technology applications are also needed in sports training and performance analysis. Firstly, within the integrated digital and intelligent PE framework, it is crucial to accurately collect and record details of students' physical postures and physiological and psychological states. Various data types are needed to support for optimizing their competitive techniques. Facing different types of sports, AI technology can help analyze which types of data need to be collected and how these data characteristics relate to students' competitive levels. In addition, these data play an important role in optimizing training plans. AI technology can perform time series correlation analysis on various types of data during students' specific training processes, which not only helps to evaluate the effectiveness of long-term training programs, but also helps teachers understand the characteristics and bottlenecks of different projects in the training process, and provide a basis for subsequent guidance. AI technology can also be used to analyze the state of team collaboration in team sports. AI-based systems can monitor and record key data of team members and analyze the performance and collaboration status of each member. Detailed statistical information and decision results can be generated as auxiliary decision-making tools for teachers. These data include position, posture, movement trajectory, passing time, success rate, running distance, etc. With the help of AI technology, the strengths and weaknesses within the team can be determined, and then targeted training plans can be formulated. At the same time, AI can simulate different tactical scenarios and evaluate their effectiveness to help teams optimize tactical strategies. With the support of AI technology, teachers and students can have a more comprehensive understanding of their teams, and the decisions made are based on data and are more scientific, which is conducive to improving the overall performance and strength of the team. In terms of team collaboration analysis, AI technology can be used to analyze the team's competitive data and optimize collaboration strategies. At the same time, more targeted combat strategies can be developed by analyzing the opponent's tactics and weaknesses. Teachers and students can be assisted in defeating the opponents. By rehearsing and predicting various scenarios that may occur in the game, suggestions can be provided for substitutions, tactical adjustments, and decision-making at critical moments. This data-driven analysis and prediction enhances the flexibility and adaptability of on-site combat and helps maximize the overall strength of the team. With the support of AI technology, on-site decision-making becomes more scientific, accurate, and efficient, thereby enhancing the team's competitiveness and chances of winning.

For students and teachers, mastering basic or simple AI technologies or developed tools or platforms can help them solve scientific research problems while improving their own abilities. But for complex technologies or products related to sports research and innovation, professional teams are required during the research and development process. Firstly, AI technology can be used to innovate teaching models and change the traditional backward method of mainly teaching and passive obedience of students. The new teaching process can be more interactive and flexible, and the establishment of this new model requires the support of technical systems and supporting products. AI can perceive and analyze the status and needs of teachers and students during the teaching process, adaptively push human-computer interaction information and adjust human-computer interaction content, build a closer communication and interaction relationship between teachers and students, and promote the effective improvement of PE teaching results. Secondly, the research and development of intelligent equipment also relies heavily on professional teams. These equipment include sportswear with AI intelligence and professional equipment for collecting data required for AI. Integrating AI functions in sportswear can not only be used to monitor and analyze the real-time status of users, but also provide users with friendly interactive functions to improve the sports experience in all aspects. In terms of data collection equipment, wearable sensors are the representative, providing vital data support for the research and development of AI technology in PE. Both the creation of such equipment and its improvement over time involve collaboration among experts from various fields and a focus on enhancing comfort and reliability. Furthermore, AI technology has great potential in the development of new sports projects. By deeply analyzing the performance, interests, physical fitness indicators and other data of students participating in various projects, we can explore the characteristics and advantages of different projects and provide reference for the design of new types of sports projects. At the same time, the development of new sports equipment with intelligent interactive functions can also improve the fun of students' sports, innovate sports models, and enrich the connotation of PE teaching. Another point to note is that with the development of science and technology and the renewal of concepts, the cross-correlation between sports disciplines and disciplines such as medicine and information science continues to deepen. AI technology can also play a key role in interdisciplinary integration research. For example, AI can combine data from medicine and sports science to explore the relationship between PE teaching and human health. In addition, AI technology can also promote the deep integration of sports physiology, medical imaging and intelligent sensing technology, and promote the development of personalized medicine and sports rehabilitation. This interdisciplinary integration not only improves the depth and breadth of research, but also promotes innovation in the fields of sports science and medicine, and provides new perspectives and methods for health management and sports training. Therefore, the application of AI technology in interdisciplinary research has significantly improved the understanding and solution capabilities of complex health problems and promoted the progress of science and technology.

Some existing general commercial AI tools or platforms can also be applied to PE teaching. For example, in terms of data analysis and management, Tableau (40–42) can help teachers create interactive dashboards and charts to analyze students' sports data, which can discover hidden trends, and thus optimize teaching strategies and exercise plans; IBM Watson (43–45) uses AI and machine learning technology to provide advanced data analysis and prediction capabilities, which can process large amounts of sports data and perform sentiment analysis, pattern recognition, and trend prediction. Decision-making and optimization in PE can be supported. In terms of health and fitness monitoring tools, Fitbit (46–48) can track users' heart rate, steps, sleep quality and exercise data in real time. Its AI functions allow teachers to monitor students' health status and adjust exercise plans based on collected data. MyFitnessPal (49–51) combines AI technology to provide exercise tracking functions, which can help users set goals, record progress, and provide personalized health advice to support students' physical management. In terms of teaching aids, ChatGPT (52–55) can be used to generate teaching content, answer student questions and provide personalized learning suggestions. Classcraft (56–58) improves student participation through gamification elements, AI technology is used to track students' behavior, and improve classroom experience. Google Classroom (59–61) provides online classroom and student interaction functions. AI functions can help teachers automatically analyze student performance, and provide personalized feedback to improve teaching efficiency. General AI tools are easier to obtain and have stable functions when used, making them easier for teachers and students to learn and use. However, when faced with professional application needs in the PE field, they cannot meet specialized application needs and require the development of dedicated AI tools or platforms.

### 3.2 The existing problems of the development of AI in PE

By analyzing the classifications of existing research in the application of AI in PE, we can better understand the current state and problems of the development of AI in PE. ChatGPT being used as an assistant in PE keiper2023artificial is an example of commercial general AI tools being employed in PE. Teachers and students can use it as reliable AI assistance in teaching and learning without needing to learn and innovate AI theories themselves. However, there are many other commercial AI tools or platforms, but existing research does not yet show evidence that these tools have been integrated into PE effectively.

Virtual sports classrooms (10, 12) and online teaching platforms (16) can not only innovate teaching models but also provide new means for interaction between teachers and students. Existing research indicates that AI applications used in these areas have already gained attention and achieved certain results. However, more and deeper technical research is still needed to construct virtual and online sports classrooms that can better meet user application needs and improve service quality. Meanwhile, although the new teaching models have their own advantages, teachers and students will experience psychological pressure when accepting new technologies and new platforms (20), which needs to be addressed.

Analyzing students' physical differences and carrying out personalized teaching (14) has also received attention, which is an example of refined and humanized PE. The differences in physical fitness and talent among students determine that the general sports training model cannot maximize the improvement of specific individual abilities. The optimization of teaching plans that adapt to students' characteristics is a manifestation of the scientific development of PE.

Monitoring students' status (15) is an important application of AI in PE, which can provide important data for the evaluation of students' learning effects. In addition to sports status monitoring, the monitoring and analysis of data such as text and audio can also be used to evaluate students' learning and teachers' teaching effects (17). Collecting data through IoT and evaluating the teaching effectiveness of teachers and students is also an effective method. For specific sports, AI-based student sports status monitoring and effect evaluation have also attracted attention. Real-time sports status monitoring can also provide a basis for teachers' interactive guidance.

Health management is an important issue that needs to be addressed in PE, including mental health. Analyzing the mental health status of college students through CNN and big data and providing early warning of health problems is a useful exploration of AI in PE. AI technology can also provide strong support in achieving a balanced development of sports health and mental health (31).

For sports training teaching, the sports theory expert system based on AI (22) has been studied to assist students in acquiring professional theories. Expert systems for specific sports have also been studied (27) to improve the effectiveness of tennis teaching. Virtual simulation teaching is used in football teaching (28). Sports training monitoring (23) has also received attention. The evaluation of students' sports conditions and the prediction of future states help improve training effectiveness. Existing research also considers the prevention (25) and treatment (26) of sports injuries, providing effective tools and methods for better protecting students' health.

In summary, we can find that the application of AI in PE is still in its early stages. The maturity and stability of the technology need to be improved, and the depth and breadth of the technology's application are still insufficient. In Figure 6, the parts of the field branches are set in red, indicating where corresponding research has been proposed. We can see that most of the existing literature is mainly focused on research conducted around the students' learning process and the teachers' teaching process. These achievements have laid the foundation for deepening the application of AI in PE. However, the relevant research is not sufficient. There are still many areas that need to be studied in each field branch, but the existing research is still sparse. There are no examples of applying AI to PE in school management. The relevant research needs more teacher teaching data and student learning and sports data as support, and there are certain difficulties in the research and expansion process. In terms of general tools, the existing AI tools have not been fully applied to PE, and how to better use commercial tools to improve the effectiveness of PE also needs further exploration. Sports research and innovation require interdisciplinary teamwork, which is also difficult in the organization and implementation process, so the existing relevant research is still insufficient.

## 4 Implications for PE teacher training

With the deepening of AI application in PE, the intelligence and digitalization of PE are gradually improving. This trend requires PE teachers to make in-depth adjustments in technology, skill, and thinking to adapt to the new teaching environment, and effectively use AI technology to improve teaching effectiveness. PE teachers need to recognize the positive role of AI technology in the transformation and upgrading of PE, and improve their cognition and acceptance of new technologies. The training of PE teachers needs to adapt to the needs of the development of smart sports and digital sports, adjust the training concepts, content and methods, improve teachers' systematic understanding of AI technology, enable them to master the use of AI tools, master common AI technologies, and have the ability to apply these technologies to solve PE problems.

### 4.1 Change of mindset

In the process of PE teacher training in the new era, improving teachers' awareness of AI technology and promoting their recognition and acceptance of the positive role of AI technology in PE transformation will help promote the wider and deeper application of AI in PE. A good training mechanism can change PE teachers' resistance to AI and alleviate their panic about unknown new things.

During the training process, teachers' confidence in AI technology can be enhanced by showing successful cases of AI application in PE, especially the specific results of how AI improves teaching effectiveness and reduces teachers' workload in these cases. At the same time, by explaining the decision-making process of AI technology, teachers can understand how AI assists teaching and increase their trust in AI systems. By explaining cases that combine AI with specific teaching goals and showing its high degree of fit with actual teaching needs, teachers' resistance to AI can be changed, and teachers can be guided to realize that AI technology will not only not replace them, but will liberate their energy, allowing them to focus more on creative teaching and personalized guidance for students.

### 4.2 Enhancing technical skills

The application of AI in PE requires teachers to possess certain technical skills. In addition to the basic theory of AI technology, the basic use of AI tools and the data collection and analysis methods need to be involved during teacher training.

With the rapid development and popularization of smart devices and wearable technology, the amount of data that can be collected and obtained in PE has increased significantly. Teachers need to master basic data analysis methods and be able to extract valuable information from a large amount of student data to optimize teaching strategies. During teacher training, it is necessary to cover the relationship between AI technology and data, enabling teachers to design data collection plans targeted at the subject objectives. At the same time, teachers should master commonly used AI models and have the ability to use these models to process data information.

During training, PE teachers need to be familiar with the use of various AI tools, including but not limited to sports status monitoring systems, intelligent teaching platforms, personalized training generators, etc. During training, teachers should master the functions and application scenarios of these tools through practical operation exercises, so as to flexibly use them in actual teaching.

### 4.3 Strengthen ethics and safety awareness

With the widespread application of AI technology in PE, issues such as ethics and data security have become increasingly important. In the process of PE teacher training, strengthening ethics and safety awareness is an important part of ensuring the effective and responsible application of AI technology in PE. To achieve this goal, the content and methods of training need to comprehensively cover multiple aspects such as data privacy, ethics and safety risk management. Schools should set up an ethics review team composed of education experts, legal experts, ethicists, psychologists and AI technology experts. During the training process, teachers need to understand the process and regulations of collaborating with the ethics review team to complete project evaluation and review.

At the same time, the importance of data privacy needs to be emphasized during the training process, and the teaching of relevant laws and regulations needs to be increased. Teachers need to understand the legal responsibilities and obligations in the process of collecting, storing, processing and sharing student-related data. In order to better help teachers achieve data privacy protection, it is necessary to increase the teaching of privacy protection tools and teach teachers how to use encryption, anonymization and de-identification and other technical means to protect the privacy of student data. When using AI tools, teachers should understand how to configure privacy protection settings to ensure that students' sensitive information is not leaked. Teachers should be clearly aware of their security responsibilities in the application of AI technology, including how to properly store student data, use AI tools correctly, and ensure the security of AI systems.

### 4.4 Promoting team collaboration and communication skills

The primary professional competency of PE teachers lies in the mastery and application of sports theory and knowledge. Most teachers only need to learn basic AI knowledge and do not need to master AI technology in depth. When completing complex AI technology applications, it is necessary to work together with multidisciplinary experts and technicians. Promoting teamwork and communication skills is essential to ensure the effective implementation of technology and optimize teaching results. Therefore, the training methods of PE teachers should be more flexible and adapt to the actual characteristics and dynamic needs of AI application in PE.

During the training process, according to specific project goals, multidisciplinary technical experts can be involved in the training system. Teachers can learn how to work with technical experts and data analysts to understand the practical application and impact of AI technology. Actual cases can also be used to show how successful interdisciplinary cooperation promotes the effective application of AI technology. Through case studies, teachers can learn how to effectively integrate expertise from different fields in teaching. Along with the actual work of teachers, flexible technical training and consulting courses can be organized regularly to solve the problems encountered by teachers in AI application, so that PE teachers and technical teams can discuss project progress, technical problems and improvement suggestions. These meetings help to solve problems in a timely manner and ensure that both parties have a clear understanding of technology applications.

## 5 Challenges and ethical considerations

Although AI technology has brought many opportunities for the scientific development of PE, it is also accompanied by a series of challenges and ethical issues, which not only affect the application effect of AI technology, but may also have a profound impact on teacher-student relationships, teaching fairness, and data privacy. Therefore, in the process of promoting the application of AI technology in PE, it is necessary to correctly handle the problems faced and enable AI technology to better serve the development of PE.

First of all, at the technical level, the effectiveness of AI systems depends largely on high-quality data. However, in PE, students' sports data, health data, etc. may be noisy, incomplete, or collected irregularly, which will directly affect the accuracy and predictive ability of AI algorithms. Teachers and schools need to formulate strict data collection standards and processes to ensure the accuracy and consistency of data. At the same time, the existing wearable devices are mostly electronic watches, and the types of reliable sensors available are still insufficient. Various types of sensor devices that can be deployed on various parts of the student's body, are comfortable to wear, and have high data collection accuracy and efficiency still need further research and development. For wearable devices that need to be worn for a long time, safety verification is also essential to avoid negative damage to the human body caused by long-term wear.

Meanwhile, AI can be an important assistant to teachers and students in PE, but teachers are still the leading role in classroom education. In addition to the high requirements of AI technology on data quality and quantity and the fairness challenges faced by the application of AI technology, AI technology also has some limitations. First, although AI technology can provide students with exercise prescriptions or provide teaching support to teachers based on the input data, it may be difficult to fully respond to the individual needs of each student, especially those with special needs or different learning styles, in the face of the huge differences in students' physical and psychological conditions. At the same time, AI technology is difficult to capture students' emotional fluctuations and cannot empathize with students, while emotional motivation plays an important role in PE. Over-reliance on AI will also weaken the social attributes of PE, such as teamwork, communication and leadership skills, which are key learning outcomes that AI cannot completely replace. Furthermore, some sports are accompanied by complex changes in body posture. Students need teachers to give real-time evaluation and feedback when participating in these complex and unpredictable sports. AI technology may not be accurate enough to provide meaningful feedback in this regard.

The application of AI in PE also brings a series of ethical and data security issues, which need to be taken seriously and resolved in the process of technology promotion. The issues of privacy protection and responsibility are the first things to consider when applying AI technology. The data information such as students' and teachers' sports postures and physical signs contain some sensitive personal data. The management of this information needs to be legal and orderly to prevent the privacy of users from being violated. At the same time, AI-based guidance on students' sports or teachers' classroom organization cannot guarantee a positive effect. If AI's suggestions have a negative impact on students' progress or health, responsibility attribution will become a serious problem. Therefore, clear guidelines for responsibility attribution must be formulated to protect the rights and interests of students and teachers. In addition, whether AI technology can be inclusive and fair is also a problem that needs attention. Applications need to accommodate the sports needs of students of different races and physical conditions. In the face of students with weak physical conditions or even disabilities, the inclusiveness and accessibility of AI technology must be ensured. Therefore, the application of AI in PE needs to be designed to adapt to different learning and physical abilities, and provide additional support for students who have difficulty using digital tools due to cognitive, sensory or motor disorders. Thus, When collecting data related to student PE, a professional review team should review the project to ensure that there are no problems in protecting students' rights and interests, informed consent, fairness and scientificity, and to ensure the morality and legality of the research. PE involves a large amount of sensitive data of students, such as health data, psychological state, etc. Some students do not want these data to be known by others, and these data are at risk of leakage during storage, processing and transmission. Schools and educational institutions need to formulate strict data privacy protection measures to ensure the security of student data. At the same time, students and parents should be informed of the use of data and its potential risks, and obtain their informed consent. A professional team is also needed to review the division of responsibilities, inclusiveness and fairness of relevant applications. Educators, policymakers and technology developers must address the above limitations and ethical issues to ensure the reasonable and effective use of artificial intelligence in physical education.

The lack of relevant laws and systems in the application of emerging technologies is also a challenge faced by the application of AI in PE, which easily leads to the abuse of technology and difficulties in defining responsibilities. Teachers' professional judgment and AI's auxiliary decision-making can both be applied to the teaching process. How to better promote the rational use of AI and prevent the abuse of AI from harming teachers or students requires relevant laws or regulations. As for existing laws and regulations, when applying AI to PE, relevant laws and regulations must be followed to ensure the legality of data collection and use. In addition, schools need to clearly define the scope and purpose of data use to prevent data abuse or use for improper purposes.

## 6 Conclusions

Through a comprehensive analysis of the existing literature on the application of artificial intelligence in sports, we can find that relevant research is still in its early stages, and existing research mainly focuses on the application exploration of student learning and teacher teaching processes. There is still a gap in research in related fields such as school sports management, innovative educational technology, and sports team collaboration analysis. Although existing research has laid the foundation for the further application of artificial intelligence in the field of sports, the maturity and stability of the technology still need to be improved, and the breadth and depth of existing literature are still insufficient. In order to promote the digital intelligent development of PE, teachers, as the main participants in sports, need to assume a more important role, actively learn artificial intelligence technology and apply it flexibly, and play a more important role in promoting the intelligent development of sports. Teacher training also needs adaptive adjustments, not only to guide teachers to master basic artificial intelligence knowledge and tool usage methods, but also to cultivate interdisciplinary collaboration capabilities, data analysis capabilities, and the ability to cope with technical challenges. At the same time, the formulation and implementation of supporting ethical and privacy protection policies, the acceptance and ability of teachers and students to use artificial intelligence technology, and ensuring the fairness and transparency of technology also need to be solved at the same time, to provide a basis for the reasonable, legal and efficient application of artificial intelligence in sports, and to ensure that artificial intelligence technology is effectively implemented and optimized in sports. This review systematically combs through the current status of AI applications in PE and points out the direction for future research. Its research significance lies in providing the potential value assessment of AI technology in PE for the education field and providing important references for education policy makers, school administrators and teachers. In addition, this review also puts forward improvement suggestions for the practical application of AI technology, promotes the further development of intelligent PE, and emphasizes the importance of interdisciplinary collaboration, technical training and ethical policies.
